# Oscillations make a self-scaled model for honeybees’ visual odometer reliable regardless of flight trajectory

**DOI:** 10.1098/rsif.2021.0567

**Published:** 2021-09-08

**Authors:** Lucia Bergantin, Nesrine Harbaoui, Thibaut Raharijaona, Franck Ruffier

**Affiliations:** ^1^ Aix-Marseille University, CNRS, ISM, Marseille, France; ^2^ CRIStAL Laboratory, CNRS UMR, 9189, University of Lille, 59650 Lille, France; ^3^ Université de Lorraine, Arts et Métiers Institute of Technology, LCFC, HESAM Université, 57070 Metz, France

**Keywords:** visual odometer, honeybees, distance flown, scaling factor, optic flow, oscillations

## Abstract

Honeybees foraging and recruiting nest-mates by performing the waggle dance need to be able to gauge the flight distance to the food source regardless of the wind and terrain conditions. Previous authors have hypothesized that the foragers’ visual odometer mathematically integrates the angular velocity of the ground image sweeping backward across their ventral viewfield, known as translational optic flow. The question arises as to how mathematical integration of optic flow (usually expressed in radians/s) can reliably encode distances, regardless of the height and speed of flight. The vertical self-oscillatory movements observed in honeybees trigger expansions and contractions of the optic flow vector field, yielding an additional visual cue called optic flow divergence. We have developed a self-scaled model for the visual odometer in which the translational optic flow is scaled by the visually estimated current clearance from the ground. In simulation, this model, which we have called SOFIa, was found to be reliable in a large range of flight trajectories, terrains and wind conditions. It reduced the statistical dispersion of the estimated flight distances approximately 10-fold in comparison with the mathematically integrated raw optic flow model. The SOFIa model can be directly implemented in robotic applications based on minimalistic visual equipment.

## 1. Introduction

It was reported in 1967 by von Frisch [1] that honeybees perform the waggle dance to convey relevant information about the distance from the hive to a food source and the direction of the corresponding flight trajectory. The nest-mates extract the relevant distance and direction information from the waggle dance and use it to find the food source themselves. However, it has not yet been established exactly how foragers assess the flight distance. It has been previously concluded that honeybees estimate this flight distance by gauging the amount of energy spent in reaching their destination [1,2]. However, recent findings have suggested that this ‘energy hypothesis’ does not actually account for the honeybees’ odometer, at least not in the case of medium distances of a few hundred metres [3]. Several authors have established that in this case, the honeybees’ odometer relies on visual cues [4–8], especially the optic flow [9] (see also [10] for a review).

The duration of the waggle run depends quite linearly on the flight distance from the hive to the food source, especially in the case of open field flights [4,8]. The slope of this linear relationship depends on some properties of the optic flow, such as those perceived when flying through a narrow highly textured tunnel [8,9] or over low-contrast lake water ripples [11]. The ventral optic flow has several properties of this kind: (i) the optic flow density, which depends on the visual contrast of the ground texture and (ii) the magnitude of the optic flow (radians per second), an angular speed which depends on both the ground speed and the ground height. Some authors have concluded that the duration of the waggle run observed in the hive depends on the accumulated ventral optic flow perceived during the forager’s flight to the food source [4,5,9,11]. Lastly, it has been suggested that honeybees may gauge the flight distance by accumulating the raw translational optic flow measured in their ventral viewfield [5–7,9,11], as described in the previous model for the visual odometer which we have referred to here as the OFacc model.

The question arises as to how mathematical integration of the raw translational optic flow (expressed in radians/s) can reliably encode a distance. It has not yet been established how a visual flight odometer fed solely with translational optic flow can be reliable, since this cue depends on both the insects’ velocity and their height of flight with respect to the ground. A pioneering biorobotic study has shown that raw mathematical integration of the optic flow does not suffice to obtain a reliable visual odometer [12]. Several visual odometric approaches involving the use of either optic flow [13] or the sparse-snapshot method [14] have been successfully tested on flying robots. All these approaches require ground height information providing the factor scaling the visual information. This scaling factor is often determined separately, using a static pressure sensor [15] or stereovision [13,14], for example. A neuroanatomical constraint model for the bee brain’s path integration process based on raw accumulated optic flow was recently tested on a mobile robot [16]: this terrestrial robot was endowed with an optic flow camera moving at an intrinsically constant height from the ground.

Horizontal and vertical oscillations have been observed during forward flight in hymenopterans in horizontal [17] and vertical tunnels [18] (see electronic supplementary material, figure S1). These movements generate contractions and expansions in the optic flow vector field, which can be quantified by the optic flow divergence. During oscillatory forward flight, the sequence of contractions and expansions is superimposed on the translational optic flow. Various optic flow cues such as translational optic flow [19] and optic flow divergence [20,21] have been used to explain insects’ visually controlled landing performances. Moreover, translational optic flow [22] and optic flow divergence [23–25] have been used to control robots’ manoeuvres. The present study was based on the previously developed visuo-motor model for honeybee flight [19,26], which includes the honeybee’s dynamics and the optic flow regulation process [22], which keeps the translational ventral optic flow constant (as observed in honeybees [27,28]).

In this paper, we present a new honeybee-inspired model for the visual odometer assessing the flight distance on the sole basis of optic flow cues. In this new model, self-induced oscillations generating optic flow divergence are used to visually gauge the depth *h*, which we have also called the ground height or the clearance from the ground. This information serves to scale the time-based integration of the concomitant translational optic flow. The present model has been called the **SOFIa** model, which stands for **S**elf-scaled **O**ptic **F**low time-based **I**ntegr**a**tion model. The SOFIa model output resulting from the scaling by the current clearance from the ground is given here in metres, whereas that of the model-based solely on the raw mathematical integration of the optic flow is given in radians. The SOFIa model was tested in simulation under a large range of flight trajectories and wind conditions, as well as over flat and irregular surfaces representing the ground.

## 2. A model for bees’ visual odometer based solely on optic flow cues

**Figure 1.**
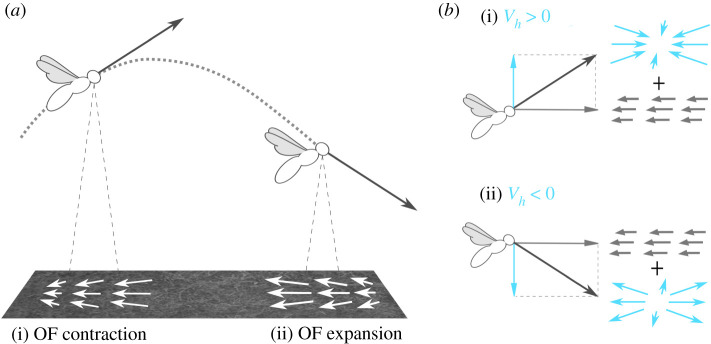
(*a*) Honeybees perform up-and-down oscillatory movements while flying forward over the ground. This process of self-oscillation generates alternating contractions and expansions of the ventral optic flow vector field, which can be quantified using the optic flow divergence cues. The optic flow divergence depends downward on the ratio *ω*_div_ = *V*_*h*_/*h*. (*b*) If the vertical velocity with respect to the ground *V*_*h*_ is positive, the optic flow divergence component will be a contraction (i); if it is negative, the optic flow divergence component will be an expansion (ii). The contraction or expansion of the optic flow is superimposed in the optic flow vector field on the translational optic flow, whose magnitude depends downward on the ratio *ω*_*T*_ = *V*_*x*_/*h*.

The SOFIa model assesses the flight distance by integrating over time the translational optic flow scaled by the current estimated height of flight $\hat{h}$. The self-oscillations performed by the simulated honeybee flying over a surface result in the superimposition of two vector field components of the optic flow (figure 1), from which the simulated honeybee can extract:

— the translational optic flow, whose magnitude depends downward on the ratio between the forward ground speed *V*_*x*_ and the height of flight *h*
  2.1 $$\omega_{T}=\frac{V_{x}}{h}\;[{\mathrm{rad}\;s}^{-1}],$$
  and
— the optic flow divergence, which depends downward on the ratio between the vertical speed *V*_*h*_ and the height of flight *h*:
  2.2 $$\omega_{\mathrm{div}}=\frac{V_{h}}{h}\;[{\mathrm{rad}\;s}^{-1}]$$

The optic flow divergence makes the clearance from the ground $\hat{h}$ observable via an extended Kalman filter (EKF) (see sections 5.3 and 5.4 of Material and methods for the state space representation of the EKF as well as for the observability analysis; see also the EKF equations S1–S7 in section S2 of electronic supplementary material). The estimated flight distances ${\hat{X}}_{\mathrm{SOFIa}}$ were determined by integrating over time an estimated linear speed, defined as the translational optic flow $\omega_{T}^{\mathrm{meas}}$ scaled by the estimated height of flight $\hat{h}$, as follows:

2.3 $${\hat{X}}_{\mathrm{SOFIa}}=\int \omega_{T}^{\mathrm{meas}}\cdot \hat{h}\;dt.$$

**Figure 2.**
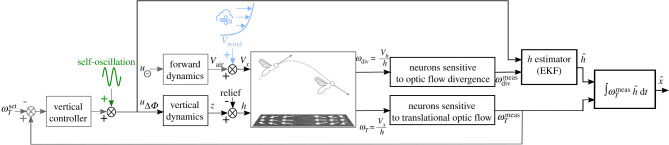
The control scheme implemented in the honeybee-inspired autopilot includes a feedback loop for vertical control, which keeps the translational optic flow constant, feeding the vertical controller with the difference between the translational optic flow perceived $\omega_{T}^{\mathrm{meas}}$ and its setpoint $\omega_{T}^{\mathrm{set}}$. The forward dynamics are modelled by a transfer function between the simulated bee’s pitch $u_{\Theta }$ and the air speed *V*_air_. The velocity of the wind is added to *V*_air_, giving the forward velocity *V*_*x*_. A height estimator (an EKF) receives (i) the wing stroke amplitude $u_{\Delta \Phi }$ as the control input (or efference copy) and (ii) the optic flow divergence $\omega_{\mathrm{div}}^{\mathrm{meas}}$ as measurements. The estimated flight distances are assessed by mathematically integrating over time the translational optic flow $\omega_{T}^{\mathrm{meas}}$ scaled by the estimated height of flight $\hat{h}$. Neurons sensitive to optic flow divergence and translational optic flow have been identified in honeybees (see Discussion section).

The honeybees’ trajectories were simulated using the results of previous modelling studies on honeybees, focusing in particular on the ventral optic flow regulator [19] and the simplified honeybees’ flight dynamics [26] (see Honeybees’ vertical dynamics in section 5.1 of Material and methods). To test the SOFIa model for the visual odometer, we simulated a honeybee performing self-controlled oscillatory movements regulating its downward translational optic flow in the presence of disturbances (figure 2). The self-scaled model for the visual odometer was first tested in simulation over flat ground in the presence of tail and head wind, and then with additional ground irregularities with various heights and slopes.

The honeybee-inspired autopilot scheme includes an optic flow feedback loop controlling the simulated honeybee’s vertical dynamics [19,26], as shown in figure 2. The vertical controller adjusts the wing stroke amplitude $u_{\Delta \Phi }$, which drives the vertical dynamics and hence the clearance from the ground (or height of flight) *h* in order to keep the ventral optic flow constant. The wing stroke amplitude $u_{\Delta \Phi }$ is the sum of the vertical controller’s output and the self-oscillatory control input (see Simulated honeybee flight parameters in section 5.2 of Material and methods). The height of flight is disturbed by the presence of irregularities on the ground. In parallel, the forward dynamics fed by the honeybee’s pitch $u_{\Theta }$ give the honeybee’s forward velocity, which can be is affected by the wind velocity (see Wind modelling in section 5.5 of Material and methods).

The translational optic flow cannot give either the ground speed or the ground height directly, but only the ratio between these two variables. This means that the use of an optic flow regulator controlling the wing stroke amplitude involves an inverse nonlinearity ($\frac{1}{x}$): the feedback loop does not linearly act on the optic flow, but rather provides the means of adjusting the denominator on which the optic flow depends, i.e. the height of flight *h* (figure 2).

## 3. Results

### 3.1. Self-oscillations make the height of flight *h* assessable whatever the wind conditions

**Figure 3.**
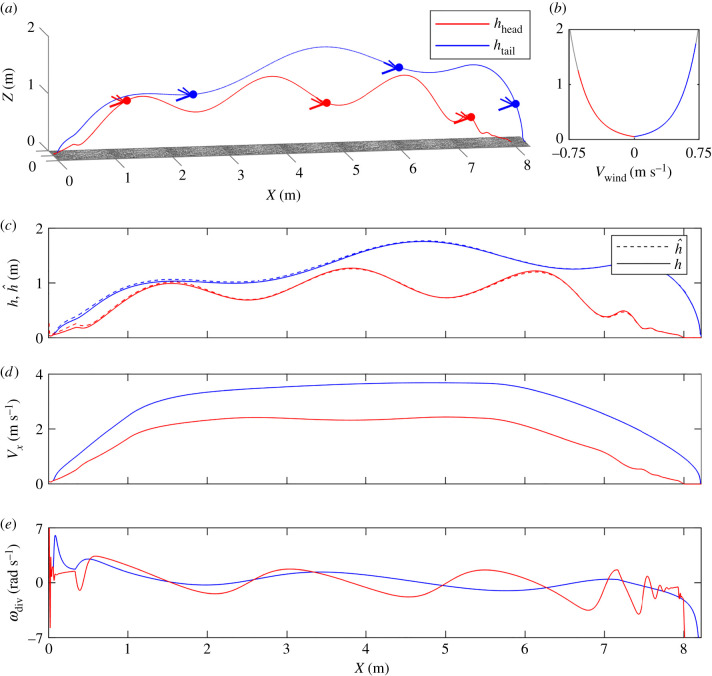
Oscillating forward flights of honeybees were simulated over a 8 m-long flat ground (see Simulated honeybee flight parameters in section 5.2 of Material and methods). (*a*) The trajectory including take-off, cruise flight and landing was simulated under tail (blue) and head (red) wind conditions. The honeybee’s pitch determined the speed and hence the height of flight *h*, in line with the optic flow regulation scheme. The change in the pitch $u_{\Theta }$ was responsible for take-off and landing. (*b*) The wind was modelled as in equation (5.7) (see Wind modelling in section 5.5 of Material and methods). Its sign depended on its direction: it was positive in the case of tail wind (blue) and negative in that of head wind (red). (*c*) The estimated height of flight $\hat{h}$ (in dashed lines) converged quickly to *h* under various initial EKF conditions. (*d*) The velocity *V*_*x*_ increased during take-off, ranged around a constant value during cruise flight, and decreased during landing. Its value during cruise flight depended on the wind conditions: it was higher in the case of tail wind (blue) and lower in that of head wind (red). (*e*) The undulating patterns of optic flow divergence were due to the vertical self-oscillatory movements. At a given optic flow setpoint, the amplitude of the optic flow divergence was greater in the case of head wind due to the honeybee being closer to the ground (see electronic supplementary material, figure S4).

Simulations of a vertically oscillating honeybee flying forward over a 8 m-long flat ground were performed. These self-oscillations generated an undulating pattern of optic flow divergence, as shown in figure 3*e*. The simulations included automatic take-off, cruise flight and landing based on the ventral optic flow regulator (see Simulated honeybee flight parameters in section 5.2 of Material and methods). Two different wind conditions (tail and head wind) were studied (figure 3*b*).

As shown in figure 3*a*, the simulated honeybee’s height of flight depended on the wind conditions. Under tail wind conditions, the simulated honeybee flew higher with respect to the ground in order to keep the perceived translational optic flow near the optic flow regulator’s setpoint. It has been previously reported that a stronger tail wind results in a higher ground speed in honeybees [29], and that flying insects (such as locusts and honeybees) ascend by tail wind and descend by head wind ([30,31], respectively). For the same reason, a head wind results in a lower value of *V*_*x*_ and hence in a lower height of flight.

Even in the presence of wind, it was possible to estimate the simulated honeybee’s clearance from the ground accurately using an EKF. As shown in figure 3*c*, the height of flight estimates $\hat{h}$ converged quickly with the ground-truth *h* and followed *h* accurately throughout the entire trajectory. The final estimation error amounted to 3.67% in the case of head wind and 5.18% in that of tail wind. $\hat{h}$ can therefore be used as a scaling factor by the SOFIa visual odometer model in order to determine the estimated flight distances accurately.

### 3.2. SOFIa odometer assesses flight distances under various conditions

**Figure 4.**
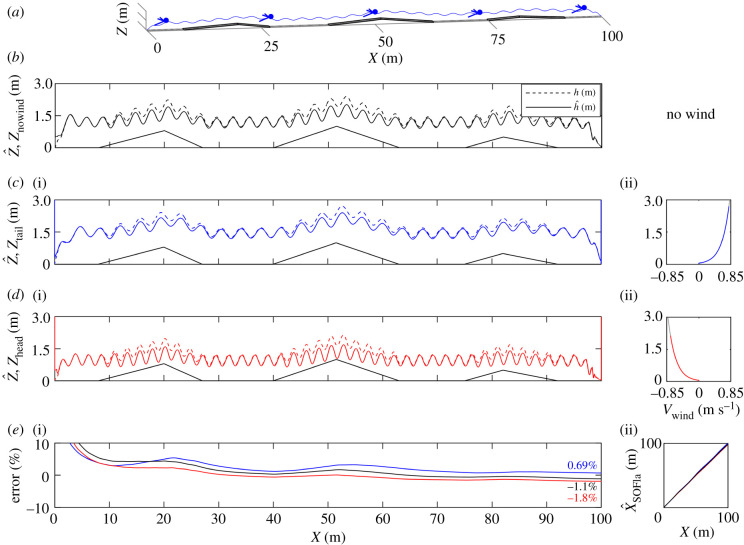
Oscillating forward honeybee flights were simulated over a 100 m-long ground including irregularities with various heights and slopes (see Simulated honeybee flight parameters in section 5.2 of Material and methods). (*a*–*d*) The simulated trajectory included take-off, cruise flight and landing under three different wind conditions: no wind (*b*) in black, tail wind (*c*) in blue, and head wind (*d*) in red. When wind was present, its sign depended on its direction: it was positive in the case of tail wind, (*c*)(ii), and negative in that of head wind, (*d*)(ii). The estimated height of flight $\hat{h}$ (in dashed lines) converged quickly with *h* under various initial EKF conditions. (*e*)(i) The error in the estimated flight distances with respect to the ground truth was normalized and expressed in %. In the absence of wind, its final value was −1.1%, under tail wind, it was 0.69%, and under head wind, it was −1.8%. (*e*)(ii) The ${\hat{X}}_{\mathrm{SOFIa}}$ estimated flight distance values were compared with the ground truth *X* throughout the entire trajectory: the estimates were found to be accurate although they were based on optic flow cues alone.

To test the robustness of the SOFIa model even in the presence of ground disturbances, simulations were performed over a 100 m-long ground surface including irregularities with various heights and slopes in the presence of wind (figure 4).

As shown in figure 4, even in the presence of multiple disturbances, including here the presence of a relief, the clearance from the ground $\hat{h}$ was still accurately estimated using the EKF thanks to the self-controlled oscillations. Again, $\hat{h}$ can be used as a scaling factor by the SOFIa visual odometer model to estimate the distance flown. Figure 4*e* gives examples of the results obtained in the estimation of ${\hat{X}}_{\mathrm{SOFIa}}$: the final estimation error here was 1.1% in the absence of wind, 0.69% under tail wind and 1.8% under head wind.

### 3.3. The SOFIa odometer is more precise than the OFacc model

The two models for the honeybee’s visual odometer (the OFacc model based solely on the raw mathematical integration of optic flow and the SOFIa model) were both tested in simulation under the same set of 630 parametric conditions in order to analyse and compare fairly the statistical distributions of the estimated flight distances. The set of simulated conditions was generated by varying: (i) the peak height of the ground relief, taking three different values of *h*_peak_ (no relief, 1 m and 2 m), (ii) the wind speed, taking seven different *k*_wind_ values ranging from −1.5 to 1.5 with a 0.5 interval, giving a full set of wind conditions ranging from head to tail wind, (iii) the translational optic flow setpoint, taking four different values of $\omega_{T}^{\mathrm{set}}$ ranging from 2 to 3.5 rad s^−1^ with a 0.3 rad s^−1^ interval and iv) the honeybee’s flight speed, taking five different time-profiles of the pitch $u_{\Theta }$, the cruising values of which ranged between 30° and 50° with an interval of 5°.

The SOFIa model’s outputs were quite accurate, giving a median distance of 104.8 m when simulated over a 100 m-long irregular surface under various wind conditions (ranging between 1.38 m s^−1^ and −1.25 m s^−1^) and relief heights (up to 2 m). The two models for the visual odometer were also simulated in a large range of heights of flight *h* and ground speeds *V*_*x*_, from 0 to 4.35 m and from 0 to 4.95 m s^−1^, respectively.

**Figure 5.**
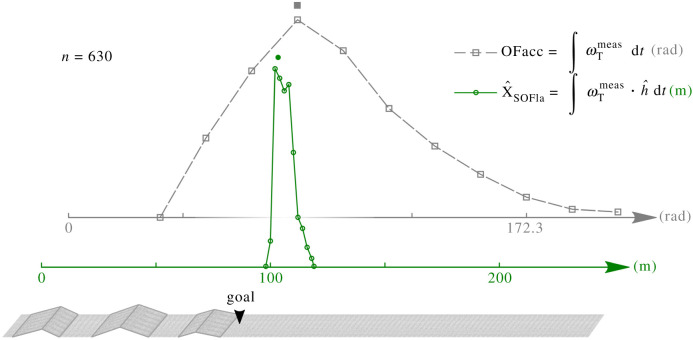
The curves give the distributions of the two models for the visual odometer simulated during a 100 m-long flight above three small hills with gentle slopes separated by flat areas, under a total number of 630 parametric conditions. The set of simulated conditions was generated by modulating (i) the peak height of the ground relief (ii) the wind speed (iii) the translational optic flow setpoint and (iv) the honeybee’s flight speed. For the sake of visual comparisons, the median value in radians of the OFacc model was aligned with the 100 m tick on the metre abscissa using the factor *k*_comparisons_ (see section 5.6 of Material and methods). The line carrying the empty circle markers denotes the relative frequency distribution given by the SOFIa model, and the line with the square markers denotes the relative frequency distribution given by the OFacc model based on the raw accumulated optic flow. The bullet symbol placed above the curves at 104.8 m gives the median value of the estimated flight distances based on the SOFIa model in comparison with the goal (the virtual food source) located 100 m away. The spread of the two models’ outputs differed significantly (Brown–Forsythe test, d.f. = 502, F: 383.66, *p*-value << 0.001). The statistical dispersion of the distribution obtained with the SOFIa model was found to be considerably smaller than that obtained with the OFacc model: the median absolute deviation (MAD) of the SOFIa model was 3.09 m, while that of the OFacc model amounted to 25.62 rad, i.e. 29.74 m after multiplication by *k*_comparisons_.

**Figure 6.**
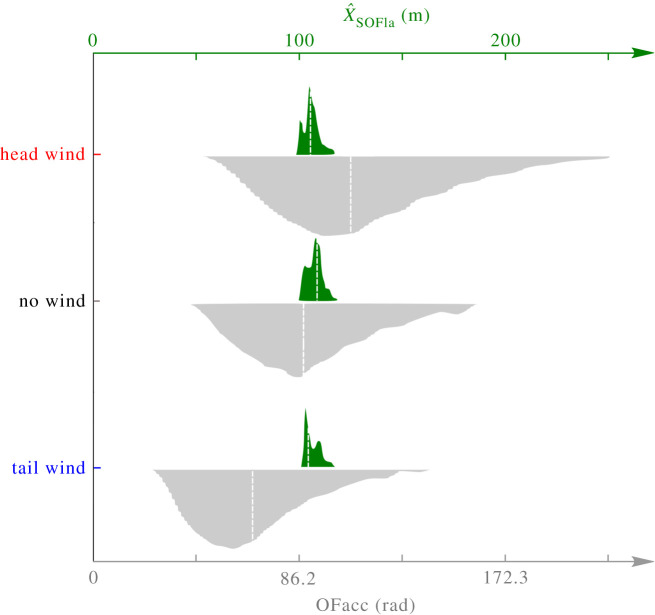
The plots give the statistical distributions obtained under the same 630 parametric conditions with the SOFIa model (in green) and the OFacc model based solely on the raw mathematical integration of the optic flow (in grey) under tail wind, no wind and head wind conditions. Under tail wind conditions, the median values of the statistical distribution obtained were 77.7 m with the OFacc model (after multiplication by *k*_comparisons_) and 103.72 m with the SOFIa model. In the absence of wind, the median values were 100.67 m with the OFacc model (after multiplication by *k*_comparisons_) and 106.67 m with the SOFIa model. Under head wind conditions, the median values obtained were 122.96 m with the OFacc model (after multiplication by *k*_comparisons_) and 104.78 m with the SOFIa model. The median values of the OFacc model outputs differed significantly among the three wind conditions (Kruskal–Wallis test, *p*-value < 10^−49^ with the OFacc model). Under the various wind conditions considered, the median values of the two visual odometer models’ outputs differed significantly (Wilcoxon test, *p*-value < 0.001, *Z* = 20.13; 11.64; 20.13, under tail wind, no wind and head wind conditions, respectively). Overall, the SOFIa model’s odometric performances were consistently more reliable regardless of the wind conditions.

The distribution of the OFacc visual odometer’s outputs was multiplied by *k*_comparisons_ after the simulation runs just to be compared statistically with that of the SOFIa model, since its output is given in *radians* and not in metres (see section 5.6 of Material and methods). As shown in figure 5, the statistical dispersion of the estimated flight distances with respect to the wind obtained with the SOFIa model differed considerably from those obtained with the OFacc model (Brown–Forsythe test, d.f. = 502, F: 383.66, *p*-value << 0.001). The median absolute deviation (MAD) of the results obtained with the SOFIa model amounted to 3.09 m, whereas that obtained with the previous model amounted to 29.74 m after multiplication by *k*_comparisons_. Figure 6 shows the statistical distributions of the outputs of the SOFIa model and those of the OFacc model under tail wind, no wind and head wind conditions. The median values given by the SOFIa model ranged between 103.72 m and 106.67 m, whereas those given by the OFacc model ranged between 77.7 m and 122.96 m after multiplication by *k*_comparisons_. The MAD of the SOFIa model was consistently lower than 3.16 m, while the MAD of the OFacc model ranged between 19.72 m and 28.71 m after multiplication by *k*_comparisons_. Our findings show that the statistical dispersion of the estimated flight distances obtained with the SOFIa model was about 10 times smaller than that of the estimates obtained with the OFacc model. Similar results were obtained upon comparing the odometric performances of the two models in terms of the honeybee’s body pitch $u_{\Theta }$, which drives its forward speed, as well as in terms of the height of the simulated relief *h*_peak_ (see figure S5.a and b in section S6 in electronic supplementary material).

At a given optic flow setpoint, the honeybee flies at a slower ground speed under head wind or low body pitch conditions and therefore takes longer to reach the food source (see figure 6 and figure S5.a in the electronic supplementary material, respectively). As a result, the OFacc model integrates this regulated ventral optic flow mathematically during a longer time and hence overestimates the flight distance. Conversely, in the case of tail wind or high body pitch, the honeybee flies at a faster ground speed and takes less time to reach the food source, and therefore the OFacc model integrates this optic flow during a shorter time, and hence underestimates the flight distance. In short, in the case of head or tail wind and low or high speed, the output of the OFacc model deviates increasingly with time from the actual distance flown by the simulated honeybee. By contrast, the output of the SOFIa model varies very little, especially depending on the forward flight speed or the wind direction or speed.

## 4. Discussion

The new model for the honeybee’s visual odometer called SOFIa presented in this study was based on biologically plausible optic flow cues. This reliable bio-plausible visual odometer can be used to assess the flight distance based on two different characteristics of the ventral vector field of the optic flow, while at the same time accounting for honeybees’ visual odometric performances.

### 4.1. *Open-field* simulation and height of flight

Here, we have presented the results obtained on simulated honeybees flying over a *virtual* open field, possibly in the presence of landforms. Under these conditions, the simulated honeybee’s ventral optic flow regulator is free to adjust its altitude without being constrained by the height of a tunnel. In tunnels, the honeybees’ height and lateral position depend on the tunnel’s geometrical configuration and on the position of the initial entrance point, while the flight speed depends on the smallest tunnel cross-section [18,27,32]. The impact of the tunnel’s geometrical configuration on the height of flight and thus on the performances of the ventral visual odometer is therefore more pronounced in narrow low-roofed tunnels. For example, the accuracy of the behaviour observed in honeybees while searching in narrow tunnels [6] and in the open field [33] differs considerably under tail wind conditions, when the mean errors recorded were −14, 4% and 3%, respectively (see table S1 in section S4 in the electronic supplementary material).

### 4.2. Reliability of the honeybee’s visual odometer documented in the literature

To reach a food source in the close vicinity, honeybees are thought to rely on several mechanisms, such as those based on visual snapshots and smell. This makes it difficult to assess the accuracy of the honeybees’ visual odometer alone, since it has not yet been established when and where on their trajectory honeybees conclude that they have reached their target. Many observations [1] have shown that honeybees are able to assess distances accurately when searching for a target containing a food source. Heran [34] has reported that honeybees signal the position of a food source in the same way whether it is placed downhill or uphill, and Bräuninger [30] has observed that honeybees can retrieve food from a source whatever the wind direction. It has also been reported that bees can sometimes fail their first attempt to find a food source immediately after attending a waggle dance [35]. Some intra- and inter-individual variability is known to exist in hymenopterans, and this has been observed in honeybees’ trajectories while they are landing on a food source [28], as well as during altitude control in a vertical tunnel [32]. To our knowledge, only two quantitative studies have been published so far on honeybees’ visual odometric estimation of the flight distance to a food source. These two behavioural studies were conducted under very different conditions, the one in narrow tunnels [6] and the other in the open field [33]. In addition, the honeybees’ trajectories were analysed using two very different methods: based on honeybees’ successive U-turns in the proximity of a target in [6], and on the truncation of the tracks when they deviated by more than 90° in [33]. These two behavioural studies have yielded substantially different results in terms of the accuracy and the statistical dispersion (see table S1 in section S4 in the electronic supplementary material for a quantitative comparison expressed in % error and relative dispersion). It is therefore difficult to make comparisons between these two behavioural studies and either the SOFIa model or the OFacc model. These comparisons might be possible only in a single case: under tail wind conditions, the accuracy of the results of the SOFIa model obtained in the *virtual* open field (0.69%) matches the accuracy observed in [33] (3%) better than that of the OFacc model (−26% after multiplication by *k*_comparisons_). Generally speaking, the distribution spread and the accuracy of the results obtained with the SOFIa model correspond to the fact that visual cues, especially optic flow cues, can reliably feed the honeybee’s visual odometer over distances of the order of a few hundred metres regardless of the trajectory taken [5,7–10].

### 4.3. Oscillations help bees to gauge their clearance from the ground

Self-controlled oscillations generate a sequence of contractions and expansions in the optic flow vector field. These contractions and expansions, which in each case can be quantified by the optic flow divergence, are superimposed on the translational optic flow component. These self-oscillatory movements make the state vector (*h*; *v*_*h*_) locally observable, and therefore the height of flight *h* can be estimated using an EKF (see sections 5.3 and 5.4 of Material and methods). In fact, an EKF is a nonlinear filter that estimates the *h*, which is also called the clearance from the ground or the depth, using the optic flow divergence and the wing stroke amplitude control signals. On this basis, the self-controlled oscillations make it possible for the ventral translational optic flow to be scaled by the clearance from the ground.

Previous experiments with honeybees flying both freely and in tunnels have shown the presence of these self-oscillatory movements. Honeybees’ self-oscillations have also been described quantitatively in narrow horizontal and vertical tunnels: the oscillation frequency ranges around 2 Hz, and the amplitude of the oscillations is approximately 3 cm in width (in narrow 12 cm-wide tunnels) [17] and 10 cm in height (in 40 cm-high tunnels) [18] (see electronic supplementary material, figure S1). Baird *et al.* [36] have recently investigated the hypothesis that honeybees flying in narrow tunnels might control their height of flight by means of sideways self-oscillations with a mean frequency of 4.7 ± 1.6 Hz. Besides hymenopterans, vertical oscillations are also known to occur in lepidopterans: one can easily observe the erratic bouncing and fluttering flight patterns of butterflies inhabiting various continents [37,38] as well as series of up and down vertical curves described by moths at a frequency of about 2 Hz [39].

The SOFIa model was tested in simulation under conditions resembling those pertaining in an open field or a wide tunnel, where honeybees seem to oscillate at lower frequencies. In all the figures presented here in the main text, the simulated honeybees oscillated with a frequency of 1 Hz and an amplitude of 35 to 55 cm, depending on the height of flight, the air speed, and the optic flow setpoint. Further experiments performed with a simulated honeybee oscillating at 2 Hz, 3 Hz and 4 Hz showed there were no significant differences in the spread of the flight distance estimates ${\hat{X}}_{\mathrm{SOFIa}}$ assessed by the SOFIa model (Brown–Forsythe test, d.f. = {3, 2516}, *p*-value = 0.899) (see section S3 and figures S2 and S3 in the electronic supplementary material).

### 4.4. Biological plausibility of the SOFIa model

In honeybees, various motion-sensitive neurons respond to specific motion patterns: (i) the velocity-tuned neurons (VTs) and some descending neurons (DNs) respond to translational optic flow [40,41] and (ii) other DNs respond to the expansion and contraction of the optic flow [42,43]. DN motion-sensitive neurons cover the ventral field of view [44] and respond to downward visual motion [45]. Honeybees are therefore sensitive to the ventral optic flow of translation, of expansion and of contraction generated by their oscillatory forward flights. The use of stereo visual cues is not biologically plausible here because flying insects lack both a binocular ventral visual field aimed towards the ground and a sufficiently large spatial resolution in their ventral visual region to be able to estimate their height of flight. Nor did stereo visual signals play a role in the case of gulls’ offshore take-off [46]. In particular, birds’ binocular visual field is rather used to guide their beaks for manipulation purposes, for example [47,48]. The optic flow-based estimator of the scale factor is a nonlinear filter (EKF) based here on (i) a nonlinear model, (ii) a control input (or called the efference copy in biological systems), and (iii) a biologically plausible sensory output (the optic flow divergence). The SOFIa model can therefore be said to be biologically plausible even if it is not entirely anatomically constrained. Nevertheless, it opens up some interesting functional perspectives for mesoscale modelling [49].

The SOFIa model would be reliable regardless of the base unit and the coding in which the information flow is processed, including those possibly used by honeybees to evaluate the scaling factor based on the flow divergence, to detect the translational optic flow, to process the efference copy (or control input) to weigh and accumulate input signals, and thus to assess the flight distance.

In conclusion, the self-scaled time-based optic flow integration model called SOFIa involves the use of a scaling factor extracted from the optic flow vector field. This scaling factor is the clearance from the ground estimated by means on an EKF based on the optic flow divergence generated by the bees’ self-oscillatory movements. Since the time-based integration of the ventral optic flow is scaled, the SOFIa model is less sensitive to changes occurring in the environment such as changes in the direction of the wind or in the trajectory taken. As shown here in the simulations and the standard deviation analysis, the estimated flight distances obtained were particularly reliable and accurate with a large range of ground surfaces and wind conditions. The SOFIa model for the visual odometer was found to be reliable even in the presence of multiple disturbances as well as changes in the simulated honeybees’ internal parameters, such as the optic flow setpoint and the flight speed. This model reduces the statistical dispersion of the estimated flight distance 10-fold in comparison with the previous model for the visual odometer based on the raw mathematical integration of the translational optic flow. Therefore, the SOFIa visual odometer model shows that their bouncing trajectory may help honeybees (i) to retrieve a food source, (ii) to return to the close vicinity of the hive and (iii) to communicate to their nest-mates a reliable flight distance between the hive and the food source.

Since the scaling was performed using a optic flow-based estimation of the clearance from the ground, the output of the SOFIa model is given in metres. In fact, the precision of the SOFIa model, combined with the dimension of its output, opens up the possibility of implementing the model directly in the field of flying robotic applications. One particularly promising future application of the SOFIa model might be in GPS-denied environments, where it would enable flying robots to assess the flight distance accurately using only minimalistic visual equipment.

## 5. Material and methods

### 5.1. Honeybees’ vertical dynamics

A simplified dynamic honeybee model was previously developed, based on behavioural studies [26]. The honeybees’ vertical dynamics were expressed in that study as follows:

5.1 $$G_{Vz}(s)=\frac{V_{z}(s)}{u_{\Delta \Phi }(s)}=\frac{K_{z}}{1+\tau_{z}s},$$

where $u_{\Delta \Phi }\;{[}^{\circ }]$ is the difference in the wing stroke amplitude in comparison with hovering, *V*_*z*_ (m s^−1^) is the vertical speed, *τ*_*z*_ = 0.22 (s) and *K*_*z*_ = 0.11. The honeybees’ forward dynamics were expressed here as follows:

5.2 $$G_{V_{\mathrm{air}}}(s)=\frac{V_{\mathrm{air}}(s)}{u_{\theta }(s)}=\frac{K_{\mathrm{surge}}}{1+\tau_{\mathrm{surge}}s},$$

where *u*_*θ*_ [°] is the honeybee’s pitch during hovering, *V*_air_ (m s^−1^) air speed, *τ*_surge_ = 0.22 (s) and *K*_surge_ = 0.10.

### 5.2. Simulated honeybee flight parameters

The controller of the ventral optic flow regulator was a PD controller with a proportional coefficient *k*_*P*_ = 15 and a derivative coefficient *k*_*D*_ = 0.3. When *h* ≥ 5 cm, the self-oscillation command *A*_osc_ sin(2*πf*_osc_) was added to the PD vertical controller output to form the wing stroke amplitude $u_{\Delta \Phi }$ control signal feeding the vertical dynamics. The 5 cm height condition made the honeybee’s take-off and landing manoeuvres suitably smooth [50]. Take-off was determined by imposing an ascending ramp on the pitch $u_{\Theta }$ between 0 m and 1 m. Landing was determined by imposing a descending ramp on $u_{\Theta }$ starting at 5.5 m in the case of simulations over an 8 m-long flat ground and at 95.5 m in that of simulations over a 100 m-long irregular ground. In figures 3 and 4, the self-oscillatory movements were simulated by a sine wave with a frequency of *f*_osc_ = 1 Hz and an amplitude of *A*_osc_ = 18°. In figures 3, 4 and S3 in the electronic supplementary material, the translational optic flow setpoint was set at 2.5 rad s^−1^.

### 5.3. State space representation of the EKF used to visually gauge $\hat{h}$

To estimate the current height of flight *h*, the EKF used (i) the downward perceived optic flow divergence as its measurement input, (ii) the model for the vertical dynamics of the simulated honeybee, and (iii) the control input signal (the wing stroke amplitude ΔΦ) regulating the vertical dynamics (see EKF equations S1–S7 in the electronic supplementary material). The continuous state space model was therefore written as follows:

5.3 $$\begin{array}{rl} \dot{x}(t) & =f\;(x(t),\Delta \Phi (t))=A.x(t)+B.\Delta \Phi (t)\\ & =\left[\begin{array}{cc} 0 & 1\\ 0 & \frac{-1}{\tau_{z}} \end{array}\right]\left[\begin{array}{c} h\\ v_{h} \end{array}\right]+\left[\begin{array}{c} 0\\ \frac{K_{z}}{\tau_{z}} \end{array}\right]\Delta \Phi \end{array}$$

and

5.4 $$y(t)=g(x(t))=\left[\frac{x_{2}(t)}{x_{1}(t)}\right]=\frac{v_{h}}{h}=\omega_{\mathrm{div}},$$

where $x=\left[\begin{array}{c} h\\ v_{h} \end{array}\right]$ is the state vector, ΔΦ is the control input and *ω*_div_ is the optic flow divergence. All the results presented here were obtained with the following initial EKF conditions: $h_{{\mathrm{EKF}}_{i}}=0.5\;m$, $V_{{\mathrm{EKF}}_{i}}=1\;{m\;s}^{-1}$.

It is worth noting that the model’s dynamics given in equation (5.3) are linear, whereas the EKF’s observation equation in (5.4) is nonlinear. We slightly adapted one EKF equation by taking the absolute value of the height of flight given in the previous state estimates in order to take into account the fact that the ground height can only be positive (see electronic supplementary material, eq. S1). In practice, this helped to achieve a much faster and more reliable convergence of the EKF estimates.

### 5.4. Self-oscillations make the scaling factor observable

The optic flow divergence and the self-controlled oscillatory movements make the system observable, i.e. the observation process based on the measurement input during a finite period of time (*t*_0_, *t*_1_) makes it possible to determine the state vector at the instant *t*_1_. To check the observability of the system, the observability rank condition was analysed. First, the observability matrix was calculated using the EKF observation equation (5.4) with respect to the model dynamics given in equation (5.3) [25]. The successive Lie derivatives of *g*(.) were then calculated. A system is observable if and only if the Jacobian function of the observability matrix is full rank. In the present case, the observability matrix was expressed as follows:

5.5 $$O=\left[\begin{array}{c} L_{\;f}^{0}(g(x(t))\\ L_{\;f}^{1}(g(x(t)) \end{array}\right]=\left[\begin{array}{c} g(x(t))\\ \frac{g(x(t)}{\partial x(t)}*f\;(x(t),u(t)) \end{array}\right]=\left[\begin{array}{c} \frac{V_{h}(t)}{h(t)}\\ \frac{-V_{h}{\left(t\right)}^{2}}{h{\left(t\right)}^{2}}+\frac{u}{h} \end{array}\right]$$

and

5.6 rank(O)=n.

Next, an analysis of *O* was performed in order to check whether its Jacobian was full-rank. The first function of the observability matrix was *y*(*t*) = *V*_*h*_/*h* = *ω*_div_ , and its second function was the first Lie derivative with respect to the dynamics −*V*_*h*_(*t*)^2^/*h*(*t*)^2^ + *u*/*h*. This result showed that the system is (locally) observable if and only if the input disturbance *u* ≠ 0, and *h* ≠ 0 and *V*_*h*_ ≠ 0. The continuous variation of the control signal *u* due to the self-induced input disturbances ensured that the values of the states *h* and *V*_*h*_ and the control signal were rarely zeroed. Therefore, the oscillatory movements made the clearance from the ground observable via the optic flow divergence.

### 5.5. Wind modelling

A logarithmic law was used to model the wind profile along the altitude [51] as follows:

5.7 $$v_{\mathrm{wind}}=k_{\mathrm{wind}}\cdot v_{0}\ln\frac{h}{h_{0}},$$

with the reciprocal of the Von Kàrmàn constant *v*_0_ = 0.2 m s^−1^, the current height *h* and the roughness height *h*_0_ = 0.05 m. In figures 3 and 4, the range of values spanned by the wind in each case is given in the coloured portion of the wind curves.

### 5.6. OFacc model and *k*_comparisons_ computation

The model for the visual odometer based on the accumulated (time-based integrated) raw translational ventral optic flow (named OFacc in this paper) gives as its output an angle expressed here in radians (and not in metres) as follows:

5.8 $$\mathrm{OFacc}=\int \omega_{T}^{\mathrm{meas}}\;dt\;(\mathrm{rad}).$$

The distribution of the OFacc model’s output was calibrated after the simulation runs to be expressed in metres only to be compared statistically with the distribution of the SOFIa model’s output. The calibration factor was called *k*_comparisons_ and was computed in order to set at 100 m the median value of the distribution of the OFacc model’s output obtained after a 100 m-simulated flight under the 630 parametric conditions:

5.9 $$k_{\mathrm{comparisons}}=\frac{100}{\mathrm{median}\{\mathrm{OFacc}\}}=1.161\;({m\;\mathrm{rad}}^{-1}).$$

### 5.7. Final flight distance estimates and flight distance error estimate

The distributions plotted in figure 5 and 6 were final flight distance estimates across combinations of simulation parameters. The final flight distance estimate was obtained at *X* = 100 m. The error (expressed as a %) was computed as follows:

5.10 $${\mathrm{err}}_{\text{\%}}=\frac{{\hat{X}}_{\mathrm{SOFIa}}-X}{X}\times 100,$$

where ${\hat{X}}_{\mathrm{SOFIa}}$ is the estimated flight distance and *X* is the ground truth.

### 5.8. Computation of the relative dispersion

The relative dispersion, which is also known as the coefficient of variation (CV), is a standardized measure of the dispersion of a probability distribution. It was expressed here as a percentage in table S1 in the electronic supplementary material. The relative standard deviation (rSD) was defined as the ratio between the standard deviation (SD) and the mean in the case of the parametric data, i.e. those based on [33]. The relative median absolute deviation (rMAD) is the ratio of the MAD to the median in the case of the non-parametric data, i.e. the present set of final odometric errors obtained with both simulated models.

### 5.9. Computer simulations

The two visual odometers were both simulated using Matlab/Simulink 2020 software. Raw data will be available online.

## Data accessibility

We provide ‘data_sim100m_frequencyComparison.xlsx’ in the electronic supplementary material, which includes all detailed simulation results of the trajectories for oscillation frequencies of 1Hz, 2Hz, 3Hz and 4Hz.
